# Complete Genome Sequence of Human Coronavirus OC43 Isolated from Mexico

**DOI:** 10.1128/genomeA.01256-16

**Published:** 2016-11-10

**Authors:** B. T. Taboada, P. Isa, M. A. Espinoza, F. E. Aponte, M. A. Arias-Ortiz, J. Monge-Martínez, R. Rodríguez-Vázquez, F. Díaz-Hernández, F. Zárate-Vidal, R. M. Wong-Chew, V. Firo-Reyes, C. N. del Río-Almendárez, J. Gaitán-Meza, A. Villaseñor-Sierra, G. Martínez-Aguilar, M. García-Borjas, D. E. Noyola, L. F. Pérez-Gónzalez, S. López, J. I. Santos-Preciado, C. F. Arias

**Affiliations:** a Instituto de Biotecnología, Universidad Nacional Autónoma de México, Cuernavaca, Morelos, México; b Colegio de Pediatría del Estado de Veracruz, Veracruz, México; c Facultad de Medicina, Universidad Nacional Autónoma de México, México D.F., México; d Hospital General de México, México D.F., México; e Hospital Pediátirco de Coyoacán, México D.F., México; f Nuevo Hospital Civil de Guadalajara “Dr. Juan I. Menchaca,” Guadalajara, Jalisco, México; g Centro de Investigación Biomédica de Occidente, IMSS, Guadalajara, Jalisco, México; h Hospital General de Durango, Durango, México; i Universidad Autónoma de San Luis Potosí, San Luis Potosí, México; j Hospital Central “Dr. Ignacio Morones Prieto,” San Luis Potosí, México

## Abstract

We report the complete genome sequence of the first Mexican human coronavirus (HCoV) OC43, obtained by new-generation sequencing and a metagenomic approach, isolated from a child hospitalized with pneumonia. The genome is closely related to the other OC43 genome sequences available, ranging from 99.8% to 98.2% nucleotide sequence identity.

## GENOME ANNOUNCEMENT

Human coronaviruses have the largest RNA genomes. Human coronavirus (HCoV)OC43 belongs to the *Betacoronavirus* genus of the *Coronaviridae* and its genome is formed by a positive-sense, single-stranded RNA of ca. 31.5 kb (1). HCoV OC43 and HCoV 229E are responsible for one-third of all common colds, infecting all age groups (2), but there have been reports of a more severe lower respiratory tract involvement (3, 4). The aim of the present study was to determine the full-length genome of the first HCoV OC43 isolated from Mexico, which was obtained from a viral metagenomic analysis of a nasal washing from a child who required hospitalization due to clinical or radiological signs of pneumonia of unknown origin, negative for commonly associated respiratory viruses and bacteria, using the xTAG Bioplex respiratory viral panel and multiplex PCR Seeplex Pneumobacter ACE detection kit, respectively (5).

Nucleic acids from the sample were extracted with the PureLink Viral RNA/DNA kit. Before extraction, the sample was treated with Turbo DNase and RNase, and a random-primer amplification of the genetic material was performed as previously described (5). The sequencing was carried out with the Illumina Genome Analyzer IIx single-end platform. A total of 12,330,418 reads were generated and 1,657,101 (13.44%) were mapped to the consensus sequence of HCoV OC43. Mapping was accomplished by SMALT v.0.7.6 (6) with default parameters (only −y set to 0.8). The whole viral genome sequence was obtained with an average coverage of 3,368×. The same genome sequence was obtained by *de novo* assembly using Velvet (7). The complete Mexican HCoV OC43 genome was composed of 30,712 nucleotides, with an average G+C content of 35.51%. The strain shows the typical OC43 organization, with the following 5′ untranslated region (UTR) (nt 1 to 199), genes: orf1ab (nt 200 to 21493), NS2a (nt 21506 to 22339), HE (nt 22354 to 23622), S (nt 23640 to 27713), NS5a (nt 27792 to 28118), E (nt 28108 to 28362), M (nt 28377 to 29069), N (nt 29079 to 30425), and I (nt 29140 to 29763), and a 3′ UTR (nt 29764 to 30713).

The phylogenetic analysis of the Mexican HCoV OC43 sequence was performed using the 90 complete genomes available at GenBank (two from England, from 1967 and 2011; 39 from the United States, one from 1960 and the others from 1985 to 2000; one from France, 2001; 44 from China, 2006 to 2012; two from Belgium, 2003 to 2004; and two from Japan, 2011). It showed overall nucleotide identities of 99.8% to 98.2% with the other OC43 genomes. Based on the phylogenetic analysis, the Mexican HCoV sequence forms a cluster with four other genomes isolated in 2012 from China (KF923903.1, |KF923897.1, KF923904.1, and KF923902.1) which, together with another cluster of 28 genomes from China (2007 to 2012), form a new clade within genotype D viruses and are located at the base of the phylogeny, while samples from United States isolated before 2000 belong to genotypes A or B.

### Accession number(s).

The complete genome sequence of the Mexican OC43 strain was deposited at GenBank under the accession number KX344031.
